# IDENTIFYING IMPORTANT AND FEASIBLE ITEMS FROM THE PREFERENCES FOR EVERYDAY LIVING INVENTORY-KOREAN (PELI-K)

**DOI:** 10.1093/geroni/igae098.2798

**Published:** 2024-12-31

**Authors:** So Young Shin, Jenny Kwon

**Affiliations:** Inje University, Busan, Republic of Korea; Miami University, Oxford, Ohio, United States

## Abstract

**Purpose:**

As a follow-up study for the translation and cultural adaptation of the Preferences for Everyday Living Inventory-Korean (PELI-K), this pilot study identified the ranking of the PELI-K items selected as most important/feasible from the original PELI-K 72 items in the Korean nursing home (NH) context.

**Methods:**

A purposive snowball sampling strategy were used. The NH stakeholder advisory panel was recruited from five unique sites in one metropolitan city in Korea: two NHs, one general hospital, one teaching hospital, and one university. A total of 34 stakeholder advisory panel, including 22 experts and 12 care consumers, conducted a self-administered, in-person survey asking to score the importance and feasibility of the 72 PELI-K items. Four answer choices were available, ranging from 0 (low) to 3 (high). The sum of the scores, which adds all the scores received from participants, was calculated to rank items. The score ranges from 3 to 102 and the higher the score, the higher the importance/feasibility.

**Results:**

The top 12 important and top 11 feasible items are reported. Items related to human basic needs such as sleeping, bathroom needs, safety, and privacy were selected as important priorities. Having regular contact with family and using electronic devices to communicate with others were the most feasible preferences.

**Conclusion:**

The results from this study will help NH providers plan and deliver care that is geared toward residents’ most salient preferences and play a huge role in creating a short version of the PELI-K.

